# Supplementary material for: Following news on social media boosts knowledge, belief accuracy and trust
Source: Nat Hum Behav. 2025 Jun 27;9(9):1833–42. doi: 10.1038/s41562-025-02205-6 (PMC12454158; doi:10.1038/s41562-025-02205-6)
Supplement: Supplementary file 1 — Section A. Treatment effects per country platform. Section B. Null effects on attitudes and interest. Section C. Breaking down the dynamic dependent variables. Section D. Compliance. Section D.1. Compliance in the French sample. Section D.2. Compliance in the German sample. Section D.3. Additional measures of compliance. Section E. Qualitative analysis of participants’ comments. Section F. Descriptives. Section G. Heterogeneous treatment effects. Section H. Attrition. Section H.1. Differential attrition. Section H.2. Non-differential attrition. Section H.3. Randomization. Section I. Exclusions and screens. Section I.1. France. Section I.2. Germany. Section J. Dynamic dependent variables. Section J.1. Current affairs knowledge. Section J.2. True claims. Section J.3. False claims. Section K. Results while controlling for news use. Supplementary Figures 1–7. [file 41562_2025_2205_MOESM1_ESM.pdf]

nature human behaviour

Supplementary information https://doi.org/10.1038/s41562-025-02205-6

# Following news on social media boosts knowledge, belief accuracy and trust

In the format provided by the authors and unedited

## Contents


**Contents** **1**

**1 Treatment effects per country-platform** **2**

**2 Null effects on attitudes and interest** **3**

**3 Breaking down the dynamic dependent variables** **3**

**4 Compliance** **3**
4.1 Compliance in the French sample . . . . . . . . 3
4.2 Compliance in the German sample . . . . . . . 3
4.3 Additional measures of compliance . . . . . . . 4

**5 Qualitative analysis of participants' comments** **4**

**6 Descriptives** **5**

**7 Heterogeneous treatment effects** **6**

**8 Attrition** **6**
8.1 Differential attrition . . . . . . . . . . . . . . . . 6
8.2 Non-differential attrition . . . . . . . . . . . . 7
8.3 Randomization . . . . . . . . . . . . . . . . . . . 7

**9 Exclusions and screens** **7**
9.1 France . . . . . . . . . . . . . . . . . . . . . . . 7
9.2 Germany . . . . . . . . . . . . . . . . . . . . . 7

**10 Dynamic dependent variables** **8**
10.1 Current affairs knowledge . . . . . . . . . . . . 8
10.2 True claims . . . . . . . . . . . . . . . . . . . . 9
10.3 False claims . . . . . . . . . . . . . . . . . . . . 9

**11 Results while controlling for news use** **9**

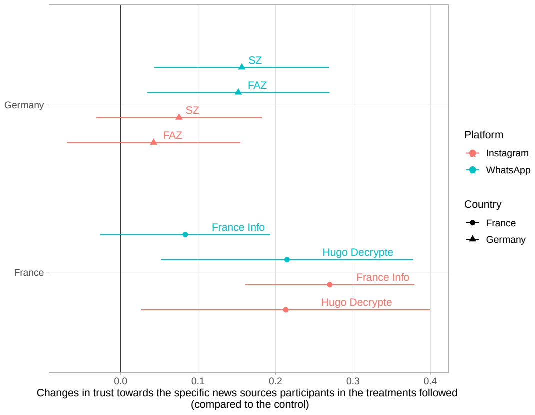


Figure 1: Treatment effects on Trust in the news accounts participants were asked to follow in the Treatments (RQ2). In France, the treatment increased trust in Hugo Décrypte ($\beta$ = .21 [.03, .40]; $\beta$ = .21 [.05, .38]) and France Info on Instagram ($\beta$ = .27 [.16, .38]) but not on WhatsApp ($\beta$ = .08 [-.03, .19]). In Germany, the WhatsApp treatments increased trust in FAZ and SZ ($\beta$ = .15 [.03, .30]; $\beta$ = .16 [.04, .27]) while the Instagram treatments did not ($\beta$ = .04 [-.07, .15]; $\beta$ = .08 [-.03, .18]).

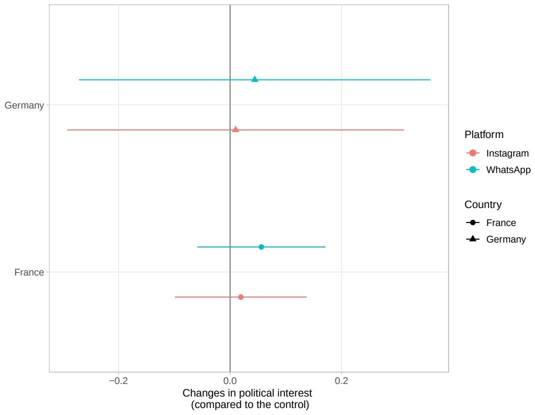


Figure 2: Treatment effects on political interest (RQ3)

## 1. Treatment effects per country-platform

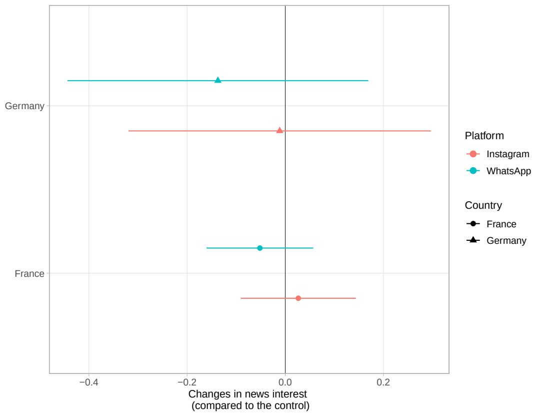


Figure 3: Treatment effects on news interest (RQ3)

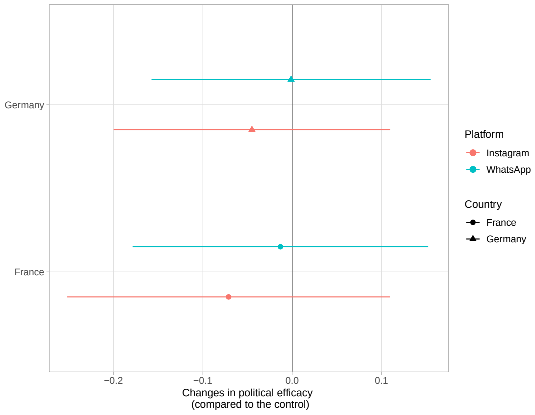


Figure 4: Treatment effect on political efficacy (RQ4)

## 2. Null effects on attitudes and interest

In the table below we report the treatment effects on the (0 to 100) feeling thermometers. These effects remain non-significant across countries and platforms.

| | b | p |
|---|---|---|
| Palestine | -0.40 | 0.59 |
| Israel | -0.11 | 0.88 |
| Ukraine | -1.04 | 0.17 |
| Russia | -0.09 | 0.90 |
| EU | -0.60 | 0.41 |
| Left-wing party | -0.11 | 0.83 |
| Center party | 0.86 | 0.18 |
| Right-wing party | 0.11 | 0.87 |

Table 1: Treatment effects on feeling thermometers

We also investigated potential effects on affective polarization, computed either as the difference between two feeling thermometers (e.g., feelings toward Ukraine minus feelings toward Russia) or as the absolute distance between feeling thermometers (e.g., "abs(Ukraine - Russia)/(Ukraine + Russia +1)"), and found no statistically significant effects.

Regarding news and political interest, as preregistered, we merged them because they were strongly correlated (Germany: r2 = .72 in Wave 1 and Wave 2; France: r2 = .70 in Wave 1 and r2 = .71 in Wave 2).

## 3. Breaking down the dynamic dependent variables

The dynamic dependent variables (DVs) are composed of items repeated in Wave 1 and 2, and new items added exclusively in Wave 2. The effect of the dynamic DVs can either be driven by the new items added in Wave 2, the repeated items present in both waves, or both. Repeated items are sometimes used to control for stable traits, such as political knowledge. However, we selected recent repeated items that participants could learn about in the next few weeks. That is, instead of asking crystallized knowledge questions, such as how often are presidential election held, we asked questions about current events, such as the name of current ministers, or when will the next European Election will be held (three months after the start of Wave 1).

In the analyses below, we break down the dynamic DVs by looking at treatment effects among repeated items exclusively, and treatment effects among new items exclusively. For current affairs knowledge, treatment effect was larger for new ($\beta$ = .06, p = .075) than repeated items ($\beta$ = .03, p = .16), but both do not reach statistical significance. For true beliefs, treatment effect was larger for new ($\beta$ = .12, p < .001) than repeated items ($\beta$ = -.02, p = .45). For false beliefs, treatment effects are small and non-significant for both new ($\beta$ = .02, p = .50) and repeated items ($\beta$ = -.01, p = .84). For awareness of true news stories, treatment effect was larger for new ($\beta$ = .10, p = .002) than repeated items ($\beta$ = -.02, p = .55). For awareness of false claims, treatment effects are small and non-significant for both new ($\beta$ = .01, p = .72) and repeated items ($\beta$ = .03, p = .29).

Overall, treatment effects tended to be stronger for new rather than repeated items. Given that for true news stories and current affairs knowledge there are more new items than repeated items, the stronger effect for new items could simply reflect greater power or precision to estimate treatment effect. It could also mean that new items were more likely to be covered in the news, and subsequently picked up by participants in the Treatments.

## 4. Compliance

To quantify compliance, two research assistants manually coded the screenshots. For each screenshot, they coded whether (i) they followed the accounts they were asked to follow and (ii) whether the notifications were activated.

We used the following procedure. The first author of the article, as well as two research assistants, coded the same 58 screenshots to agree on coding rules. Then, the research assistants coded the same 398 screenshots to estimate agreement between them. Coders gave screenshots the same ratings 93.97% of the time (935/995) – chance would be 50% given that ratings could only take two values (0 or 1). Then, one research assistant coded the remaining screenshot in the French sample and another research assistant coded the remaining screenshot in the German sample.

In the sections below, we provide some additional information about compliance based on self-reported questions in Wave 2.

### *4.1. Compliance in the French sample*

Among participants in the Treatment, when asked to write in an open text box which news source they were asked to follow in the previous wave, 78% of participants mentioned France Info and 74% mentioned Hugo Décrypte. Only 19% of participants mentioned neither, while 73% of participants mentioned both.

When asked which specific news source they follow from a list of sources, in the WhatsApp Treatment, 87% of participants selected France Info and 90% selected Hugo Décrypte, while in the Instagram Treatment, 86% of participants selected France Info and 89% selected Hugo Décrypte.

In the Treatments, 82 participants (out of 873) reported having unfollowed the news accounts. In the Controls, 91 (out of 822) reported having unfollowed the non-news accounts. Among participants who reported having unfollowed accounts, 19 reported never having followed them, 27 having unfollowed them right after the screenshots, and the rest some time later. These proportions are similar across Treatments and Controls.

### *4.2. Compliance in the German sample*

Among participants in the Treatments, when asked to write in an open text box which news source they were asked to follow in the previous wave, 74% of participants mentioned FAZ and 75% mentioned SZ. Only 19% of participants mentioned neither, and 68% of participants mentioned both.

When asked which specific news source they follow from a list of sources, in the WhatsApp Treatment, 85% of participants selected FAZ and 85% selected SZ, while in the Instagram

Treatment, 88% of participants selected FAZ and 88% selected SZ.

In the Treatments, 140 participants (out of 857) reported having unfollowed the news accounts. In the Controls, 138 (out of 843) reported having unfollowed the non-news accounts. Among participants who reported having unfollowed accounts, 12 reported never having followed them, 40 having unfollowed them right after the screenshots, and the rest some time later. These proportions are similar across Treatments and Controls.

### *4.3. Additional measures of compliance*

In Wave 2, participants in the Treatments were more likely to report having received news notifications on their phones in the past week than participants in the Controls. In wave 1, 46% of participants in the Treatment and 49% of participants in the Control reported having received news notifications on their phones, while in wave 2 this percentage goes up to 70% in the treatment and 51% in the Control. These differences are statistically significant (b = .20, p < .001).

Self-reported news consumption on WhatsApp significantly increased between waves among participants in the WhatsApp Treatment compared to participants in the WhatsApp Control (b = .78, p < .001). In Wave 1, WhatsApp news consumption was slightly higher in the Control than in the Treatment (by .04), whereas in Wave 2 WhatsApp news consumption is much higher in the Treatment than in the Control (by .77).

Self-reported news consumption on Instagram significantly increased between waves among participants in the Instagram Treatments compared to participants in the Instagram Controls (b = .44, p < .001). In Wave 1, Instagram news consumption was slightly higher in the Treatment than in the Control (by .01), whereas in Wave 2 Instagram news consumption was much higher in the Treatment than in the Control (by .45).

While self-reported news consumption on WhatsApp and Instagram increased, general news consumption (i.e., not platform specific), did not significantly in Wave 2 for participants in the treatment compared to participants in the control (b = .03, p = .43).

In Wave 2, participants in the Treatments were more likely to report that social media is their main source of news (21.9% in Wave 1 and 25.1% in Wave 2) compared to participants in the Control (21.3% in Wave 1 and 20.4% in Wave 2; b = .04, p < .001).

## 5. Qualitative analysis of participants' comments

Few participants left a comment at the end of Wave 2 in the open-ended-text box (192 in France and 190 in Germany). The majority of these comments are participants expressing gratitude (e.g., "thank you"), saying they have no comments or that they enjoyed the survey. In this section, we share some of these comments as well as our impressions of the comments. In short, many participants reported having enjoyed following the news account and said they would continue to follow them in the future. A few participants mentioned having discovered the news functionality on WhatsApp and the news on Instagram. In Germany, many complained that some articles were behind paywalls, while in France a participant complained about the 'youthful' information style used by France Info.

Many participants mentioned having enjoyed following these news account on social and plan to continue following them in the future.

"A useful survey for someone like me who doesn't follow the news. I'll keep one of the 2 accounts to keep myself informed a little." – *French participant in the Instagram treatment.*

"I didn't know about these two WhatsApp channels, but they're pretty reliable and their info overlaps. So I'll keep following them." – *French participants in the WhatsApp treatment.*

"Interesting and entertaining. I will continue to follow the two accounts because I like them." – *German participant in the Instagram treatment.*

"Very interesting study - I will continue to follow the accounts." – *German participant in the Instagram treatment.*

Some participants mentioned having discovered the news functionality on WhatsApp and Instagram because of the experiment.

"Thanks to you, I've learned that it's possible to follow accounts on WhatsApp. I was only using the instant messaging function" – *French participant in the WhatsApp control.*

"Thank you very much for the survey! I didn't know this option existed and I'm very excited to receive more sensible news, unlike what I see on Instagram." – *German participant in the WhatsApp treatment.*

In Germany some participants complained that the articles were behind paywalls:

"It's a pity that one has to pay to read the articles." – *German participant in the Instagram treatment.*

One French participant mentioned that the tone used by France Info journalists was too oriented towards young people. This is an interesting point because while both Hugo Décrypte and France Info target young audiences on social media, only France Info sometimes use an informal "youthful" style.

"I'm thinking of continuing to follow a news account. But I don't like the "youthful" tone of France Info, which starts some articles by saying djadja, which isn't a French word." – *French participant in the WhatsApp treatment.*

## 6. Descriptives

These descriptives are the predicted values from the statistical models of the main analyses (i.e., the intent-to-treat analyses adjusting for demographics, etc.).

| | Mean | SD |
|---|---|---|
| **Wave 2 True claims** | | |
| Control | 5.12 | 1.02 |
| Treatment | 5.22 | 1.03 |
| **Wave 2 False claims** | | |
| Control | 3.60 | 1.14 |
| Treatment | 3.64 | 1.15 |
| **Wave 1 True claims** | | |
| Control | 5.72 | 1.25 |
| Treatment | 5.71 | 1.27 |
| **Wave 1 False claims** | | |
| Control | 3.70 | 1.29 |
| Treatment | 3.76 | 1.31 |

Table 2: Means and standard deviations of True and False news stories across countries in W1 and W2 by Control and Treatment conditions. Note that in Wave 1 there are 2 true news stories and 2 false news stories, whereas in Wave 2 there are 7 true news stories and 4 false news stories. The accuracy scale goes from 'Completely false' [1] to 'Completely true' [8].

*Current affairs knowledge*

In Wave 1, participants in the control and the treatment had respectively 1.47 and 1.46 correct responses – the minimum being 0 and the maximum 3.

In Wave 2, participants in the control and the treatment had respectively 2.87 and 2.97 correct responses – the minimum being 0 and the maximum 7.

*Awareness of true claims*

In Wave 1, participants in the control and the treatment reported being aware of, respectively, 1.15 and 1.12 true claims – the minimum being 0 and the maximum 2.

In Wave 2, participants in the control and the treatment reported being aware of, respectively, 3.34 and 3.45 true claims – the minimum being 0 and the maximum 7.

*Awareness of false claims*

In Wave 1, participants in the control and the treatment reported being aware of, respectively, 0.32 and 0.31 false claims – the minimum being 0 and the maximum 2.

In Wave 2, participants in the control and the treatment reported being aware of, respectively, 1.01 and 1.03 false claims – the minimum being 0 and the maximum 4.

*Additional measures of discernment and bias*

When looking at additive discernment (meanTrue - meanFalse):
In Wave 2, in the control, true claims were rated 1.52pts higher than false claims.
In Wave 2, in the treatment, true claims were rated 1.58pts higher than false claims.

When looking at multiplicative discernment (meanFalse/meanTrue):
In Wave 2, in the control, true claims were rated 1.43 times higher than false claims.
In Wave 2, in the treatment, true claims were rated 1.42 times higher than false claims.

When looking at truth-bias ((maxTrue - meanTrue) - (meanFalse - minFalse)):
In Wave 2, in the control, participants showed a small skepticism bias, and rated false news as more false than they rated true news as true (by 0.28pts).
In Wave 2, in the treatment, participants showed a small skepticism bias, and rated false news as more false than they rated true news as true (by 0.14pts).

*Trust in the news*

In Wave 1, participants in the control and the treatment reported trusting the news and journalists, respectively, by 3.69 and 3.61 (3 represents 'little trusting' while 4 represents 'fairly trusting') – the minimum being 1 and the maximum 6.

In Wave 2, participants in the control and the treatment reported trusting the news and journalists, respectively, by 3.69 and 3.74 – the minimum being 1 and the maximum 6.

*Political efficacy*

In Wave 1, the political efficacy of participants in the control and the treatment was, respectively of, 3.41 and 3.40 – the maximum being 5 and the minimum 1.

In Wave 2, the political efficacy of participants in the control and the treatment was, respectively of, 3.40 and 3.38 – the maximum being 5 and the minimum 1.

*News and political interest*

In Wave 1, participants in the control and the treatment reported being interested in the news and politics, respectively, by 4.68 and 4.69 (4 represents 'Fairly interested' while 5 represents 'Very interested') – the minimum being 1 and the maximum 6.

In Wave 2, participants in the control and the treatment reported being interested in the news and politics, respectively, by 4.71 and 4.71 – the minimum being 1 and the maximum 6.

*Feeling informed*

In Wave 1, participants in the control and the treatment reported feeling informed about current events and politics, respectively, by 3.88 and 3.84 (4 represents 'Fairly informed' while 3 represents 'Slightly informed') – the minimum being 0 and the maximum 6.

In Wave 2, participants in the control and the treatment reported feeling informed about current events and politics, respectively, by 3.90 and 3.87 – the minimum being 0 and the maximum 6.

## 7. Heterogeneous treatment effects

We found very few statistically significant heterogeneous treatment effects, and the ones that reached statistical significance were barely significant and would not be considered significant after correcting for multiple comparisons. Indeed, we have five dependent variables multiplied by seven potential moderators, yielding 35 comparisons – and when looking at platform and country-specific effects the number of comparisons jumps to 140.

We report the main heterogeneous treatment effects on OSF in the 'Stats' folder.

## 8. Attrition

Below we investigate attrition and focus on the 309 German and the 325 French participants who completed the first wave but not the second wave. In both countries, the distribution of these participants across conditions is similar to the distribution of participants who finished both waves (the largest difference being of 3.7pp).

### *8.1. Differential attrition*

We tested for differential attrition across Controls/Treatments for each dependent variable, as well as demographic variables, in each country.

Overall, we tested 24 potential cases of differential attrition and found zero statistically significant effect. Below we report two cases that are close to statistical significance. On OSF, we report all 24 cases in the 'Attrition' folder.

- In France, Control participants with lower discernment in Wave 1 were slightly more likely to drop out in Wave 2, whereas it was not the case for participants in the Treatments (p = .054).

- In France, Control participants with lower trust in the news in Wave 1 were slightly more likely to drop out in Wave 2, whereas it was not the case for participants in the Treatments (p = .07).

Overall, we do not believe that these two 'almost significant' cases of differential attrition (out of 24) are particularly problematic for three reasons. First, we measured all dependent variables before and after the treatment, and accounted for pre-treatment levels in all analyses (as we compare pre- and post-changes within individuals between conditions). This allows us to account for individual baseline differences and isolate the

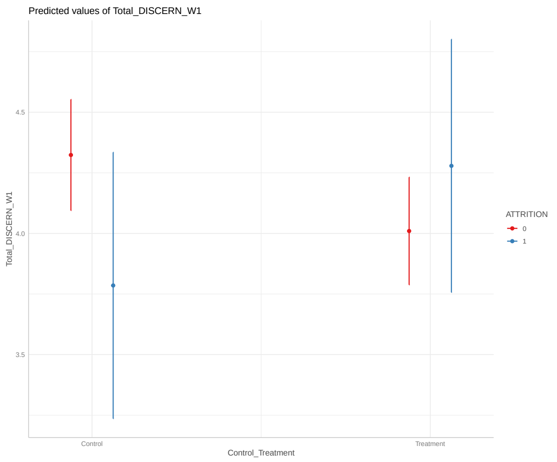


Figure 5: Discernment in Wave 1, in France, among participants who completed both waves and those who dropped out in Wave 2.

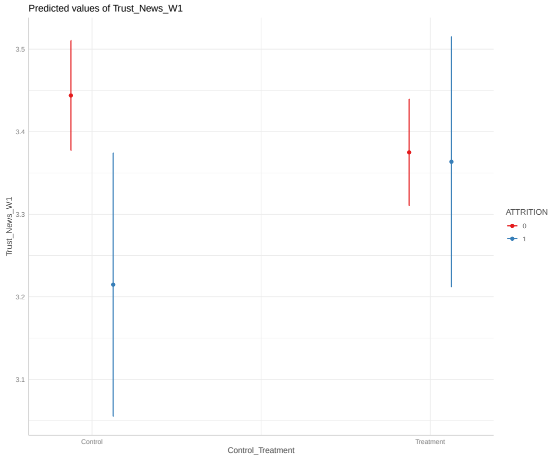


Figure 6: Trust in the news in Wave 1, in France, among participants who completed both waves and those who dropped out in Wave 2.

treatment effect more accurately. Second, our findings are consistent across France and Germany, even though we find no evidence of differential attrition in Germany. These similarities are particularly striking for trust in the news. Third, the direction of differential attrition in France is such that, in Wave 1, participants in the Controls who completed both waves are slightly more trusting and discerning than those in the Treatment, which runs counter to our hypotheses. This implies that any potential bias from attrition would likely reduce the observed treatment effect rather than inflate it, making our findings more conservative.

### 8.2. Non-differential attrition

We observed clear cases of non-differential attrition – i.e., differences in participants who completed Wave 1 but not Wave 2 (compared to participants who completed both waves) that are similar across Controls/Treatments.

In France, in Wave 1, participants who completed both waves were slightly more trusting of the news (b = .12, p = .06), older (b = -3.12, p < .001), more knowledgeable (b = .22, p < .001), more aware of true news stories (b = .15, p < .001), more interested in the news (b = .19, p < .001) and politics (b = .21, p = .01). Crucially, these differences are similar across Controls/Treatments.

In Germany, in Wave 1, participants who completed both waves were more knowledgeable (b = .20, p < .001), more likely to be women (b = .09, p < .001), and younger (b = -5.09, p < .001). These differences are similar across Controls/Treatments.

Non-differential attrition is not problematic for causal inference because it does not threaten randomization - i.e., the balance between Controls/Treatments is preserved. As a result, the estimated treatment effects remain unbiased. Although the reduced sample size due to attrition decreases the precision of our estimates, it does not threaten internal validity.

### 8.3. Randomization

OLS regressions showed that participants in the treatments did not significantly differ from participants in the controls in terms of age (FR: p = .85, GER: p = .44), gender (FR: p = .25, GER: p = .54) and education (FR: p = .78, GER: p = .54).

Note that in France participants in the WhatsApp branches had higher education than participants in the Instagram branches (b = .37, p < .001). In Germany, there were more women in the WhatsApp branches than in the Instagram branches (b = .08, p = .001). These differences are not problematic for causal inference as they are between branches (WhatsApp *vs* Instagram) and not between conditions (control *vs* treatment). Indeed, participants were allocated to the WhatsApp or Instagram branches based on their reported platform use, and were later randomly allocated to control *vs* treatment conditions within their branch.

| | Screenshot uploaders | | Non-uploaders | |
|---|---|---|---|---|
| | Control | Treatment | Control | Treatment |
| Instagram | 23.5 | 25.3 | 22.6 | 24.9 |
| WhatsApp | 24.8 | 26.4 | 24.3 | 28.2 |

Table 3: Percentage of participants per condition in France, divided by 'screenshot uploaders' (i.e., participants who finished Wave 1) and 'non-uploaders' (i.e., participants who almost finished Wave 1 but did not upload a screenshot)

## 9. Exclusions and screens

### 9.1. France

In France, in Wave 1, a total of 8051 participants took the survey.

2097 participants did not pass the initial screens and were excluded at the very beginning of the survey because they did not report having a WhatsApp or an Instagram account (715), reported never using WhatsApp and Instagram (138), or were already following one of the social media accounts (1244).

2024 participants passed the screens but voluntarily ended the survey on the second consent form, when being told that they would have to follow two social media accounts on WhatsApp/Instagram for two weeks. Most of these participants reported not wanting to follow new accounts on social media for two weeks – and only 17% reported that the compensation was too low.

1909 participants passed the screens and filled out both consent forms but did not finish the survey. Most of them (1731) left the survey at the very end when asked to follow the accounts and upload the screenshots. These participants were not re-contacted in Wave 2 as uploading the screenshots was a necessary condition to finish the survey and be eligible for Wave 2. The distribution of these participants across conditions is similar to the distribution of participants who finished the first wave. There is no sign of differential attrition across Controls/Treatments.

### 9.2. Germany

In Germany, in Wave 1, a total of 8009 participants took the survey.

1420 participants did not pass the initial screens and were excluded at the very beginning of the survey because they did not report having a WhatsApp or an Instagram account (225), reported never using WhatsApp and Instagram (149), or were already following one of the social media accounts (1046).

2859 participants passed the screens but voluntarily ended the survey on the second consent form, when being told that they would have to follow two social media accounts on WhatsApp/Instagram for two weeks. Most of these participants reported not wanting to follow new accounts on social media for two weeks – and only 15% reported that the compensation was too low.

1721 participants passed the screens and filled out both consent forms but did not finish the survey. Most of them (1611) left the survey at the very end when asked to follow the accounts and upload the screenshots. These participants were not

| | Screenshot uploaders | | Non-uploaders | |
|---|---|---|---|---|
| | Control | Treatment | Control | Treatment |
| Instagram | 24.7 | 26.5 | 18.6 | 20.4 |
| WhatsApp | 24.6 | 24.1 | 30.5 | 30.5 |

Table 4: Percentage of participants per condition in Germany, divided by 'screenshot uploaders' (i.e., participants who finished Wave 1) and 'non-uploaders' (i.e., participants who almost finished Wave 1 but did not upload a screenshot)

re-contacted in Wave 2 as uploading the screenshots was a necessary condition to finish the survey. The distribution of these participants across Control/Treatment is similar to the distribution of participants who finished the first wave. There is no sign of differential attrition across Controls/Treatments. However, participants in the WhatsApp conditions were more likely to drop out than participants in the Instagram conditions. Suggesting that in Germany participants may have struggled to follow the accounts on WhatsApp. Such differential attrition between WhatsApp/Instagram groups is not problematic for causal inference given that it does not impede the Controls/Treatments randomization – i.e., the WhatsApp Treatments are compared to the WhatsApp Controls.

## 10. Dynamic dependent variables

### *10.1. Current affairs knowledge*

For all the current affairs knowledge questions, participants were instructed 'If you don't know, simply check the answer "I don't know." Do not look up the answer online, we want to know what you think!' and were offered the option 'I don't know'. We randomized the order of the responses - except for the question on the European election and Nikki Haley. 'Don't know' was always the last option.

- Who is the current Minister of National Education and Youth? (W1 and W2 FRANCE)
o Nicole Belloubet (correct)
o Rachida Dati
o Amélia Oudéa-Castera
o Gabriel Attal
o Jean-Michel Blanquer
o Élisabeth Borne

- Who is the current Minister of Defense? (W1 and W2 GERMANY)
o Boris Pistorius (correct)
o Olaf Scholz
o Christine Lambrecht
o Annegret Kramp-Karrenbauer
o Wolfgang Schmidt
o Robert Habeck

- When will the next elections for the members of the European Parliament take place? (W1 and W2)
o In May 2024
o In June 2024 (correct)
o In July 2024
o In May 2025
o In June 2025
o In July 2025

- What is the southernmost city in the Gaza Strip, at the border with Egypt? (W1 and W2)
o Rafah (correct)
o Gaza City
o Jabalia
o Khan Yunis
o Beit Lahiya
o Deir al-Balah

- Which country has just become the 32nd member of NATO? (W1 and W2)
o Sweden
o Norway
o Finland (correct)
o Ukraine
o Georgia
o Bosnia and Herzegovina

- To facilitate humanitarian aid to Gaza, Joe Biden announced that the United States will build: (W2)
o A port (correct)
o An airport
o A highway
o A hospital
o A school
o A supermarket

- Who is Nikki Haley? (W2)
o She is the Republican candidate for the U.S. presidential election.
o She is the Democratic candidate for the U.S. presidential election.
o She is the independent candidate for the U.S. presidential election.
o She was a Republican candidate in the U.S. primaries but has since withdrawn. (correct)
o She was a Democratic candidate in the U.S. primaries but has since withdrawn.
o She was an independent candidate in the U.S. primaries but has since withdrawn.

- Which party voted against the aid plan for Ukraine in the National Assembly? (W2 FRANCE)
o National Rally (Rassemblement National) (correct)
o Renaissance
o Unsubmissive France (La France Insoumise)
o The Republicans (Les Républicains)
o Democratic Movement (MoDem)
o Horizons

- Which country has recently added the right to abortion to its constitution? (W2 GERMANY)

o France (correct)
o Sweden
o United Kingdom
o USA
o Poland
o Italy

*10.2. True claims*

- The USA has more than twice vetoed a draft UN Security Council resolution calling for an immediate ceasefire in Gaza. (W1 and W2)

- Russian opposition politician Alexei Navalny died at the age of 47 in prison. (W1 and W2)

- The US House of Representatives passed a law to ban TikTok on American territory. (W2)

- Once again, the Union failed in the Bundestag with a motion to enforce the delivery of 'Taurus' cruise missiles to Ukraine. (W2 GERMAN)

- "Macron wants to enshrine the notion of consent in French rape law." (W2 FRANCE)

- The United Kingdom has developed a laser called Dragon Fire that can hit a coin from a kilometer away. (W2)

- A man received 217 COVID vaccinations. (W2)

- Princess Kate Middleton edited and published a photo of herself and her children. (W2)

*10.3. False claims*

- Some Palestinian victims are actually 'crisis actors' pretending to be severely injured. (W1-2)

- NATO had promised Russia not to expand after the Cold War. (W1-2)

- Ukraine has been committing genocide in the Donbass region for years, and Russia had to intervene to protect the population. (W2)

- The number of deaths in Gaza has been exaggerated or even completely fabricated. Israel killed far fewer than 30,000 Palestinians. (W2)

## 11. Results while controlling for news use

In this section, we report the main results while controlling for self-reported news use in Wave 1.

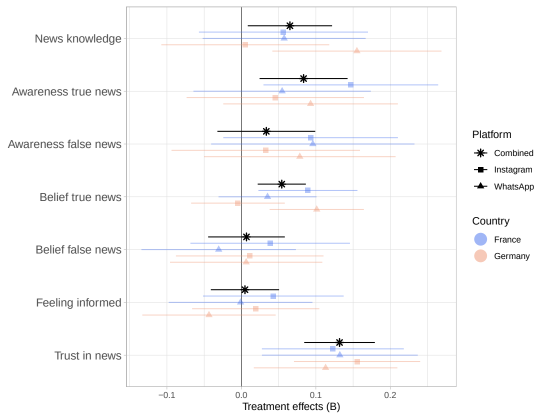


Figure 7: Main effects while controlling for news use.
