# Supplementary material for: Following news on social media boosts knowledge, belief accuracy and trust
Source: Nat Hum Behav. 2025 Jun 27;9(9):1833–42. doi: 10.1038/s41562-025-02205-6 (PMC12454158; doi:10.1038/s41562-025-02205-6)
Supplement: Supplementary file 2 — Reporting Summary [file 41562_2025_2205_MOESM2_ESM.pdf]

nature portfolio

Corresponding author(s): Magdalena Wojcieszak

Last updated by author(s): Feb 20, 2025

# Reporting Summary

Nature Portfolio wishes to improve the reproducibility of the work that we publish. This form provides structure for consistency and transparency in reporting. For further information on Nature Portfolio policies, see our Editorial Policies and the Editorial Policy Checklist.

## Statistics

For all statistical analyses, confirm that the following items are present in the figure legend, table legend, main text, or Methods section.

| n/a | Confirmed | |
|---|---|---|
| ☐ | ☒ | The exact sample size (*n*) for each experimental group/condition, given as a discrete number and unit of measurement |
| ☐ | ☒ | A statement on whether measurements were taken from distinct samples or whether the same sample was measured repeatedly |
| ☐ | ☒ | The statistical test(s) used AND whether they are one- or two-sided<br>*Only common tests should be described solely by name; describe more complex techniques in the Methods section.* |
| ☐ | ☒ | A description of all covariates tested |
| ☐ | ☒ | A description of any assumptions or corrections, such as tests of normality and adjustment for multiple comparisons |
| ☐ | ☒ | A full description of the statistical parameters including central tendency (e.g. means) or other basic estimates (e.g. regression coefficient) AND variation (e.g. standard deviation) or associated estimates of uncertainty (e.g. confidence intervals) |
| ☐ | ☒ | For null hypothesis testing, the test statistic (e.g. *F*, *t*, *r*) with confidence intervals, effect sizes, degrees of freedom and *P* value noted<br>*Give P values as exact values whenever suitable.* |
| ☒ | ☐ | For Bayesian analysis, information on the choice of priors and Markov chain Monte Carlo settings |
| ☒ | ☐ | For hierarchical and complex designs, identification of the appropriate level for tests and full reporting of outcomes |
| ☐ | ☒ | Estimates of effect sizes (e.g. Cohen's *d*, Pearson's *r*), indicating how they were calculated |

*Our web collection on statistics for biologists contains articles on many of the points above.*

## Software and code

Policy information about availability of computer code

| | |
|---|---|
| Data collection | We used Qualtrics to implement the survey |
| Data analysis | We used R and R studio to conduct the statistical analyses |

For manuscripts utilizing custom algorithms or software that are central to the research but not yet described in published literature, software must be made available to editors and reviewers. We strongly encourage code deposition in a community repository (e.g. GitHub). See the Nature Portfolio guidelines for submitting code & software for further information.

## Data

Policy information about availability of data

All manuscripts must include a data availability statement. This statement should provide the following information, where applicable:

- Accession codes, unique identifiers, or web links for publicly available datasets
- A description of any restrictions on data availability
- For clinical datasets or third party data, please ensure that the statement adheres to our policy

The pre-registrations, materials, R scripts, and the data to replicate the findings are publicly available on OSF at: https://osf.io/8tzd2/

## Research involving human participants, their data, or biological material

Policy information about studies with human participants or human data. See also policy information about sex, gender (identity/presentation), and sexual orientation and race, ethnicity and racism.

| | |
|---|---|
| Reporting on sex and gender | We only asked Gender in the survey and report no gender analysis in the manuscript |
| Reporting on race, ethnicity, or other socially relevant groupings | we did not measure race/ethnicity |
| Population characteristics | In 2024, between March 1 and March 11, we recruited 2,009 German and 2,021 French participants via the mar- ket research company Bilendi. Participants were recontacted between March 18 and 28, 1,700 German participants (985 women, mean age = 40.5 (12.5), median education = A two- year college degree, 38% had a bachelor degree or more), and 1,695 French participants completed the second wave (1,042 women, mean age = 44.1 (11.5), median education = finished high school, 31% had a bachelor degree or more). The median distance between waves was 14 days (M = 13.4, SD = 1.74). InAppendix I we report all exclusions. |
| Recruitment | They were recruited via a Survey company called Bilendi.<br><br>Appendix I.1. France<br>In France, in Wave 1, a total of 8051 participants took the survey.<br>2097 participants did not pass the initial screens and were excluded at the very beginning of the survey because they did not report having a WhatsApp or an Instagram account (715), reported never using WhatsApp and Instagram (138), or were already following one of the social media accounts (1244).<br>2024 participants passed the screens but voluntarily ended the survey on the second consent form, when being told that they would have to follow two social media accounts on What- sApp/Instagram for two weeks. Most of these participants re- ported not wanting to follow new accounts on social media for two weeks – and only 17% reported that the compensation was too low.<br>1909 participants passed the screens and filled out both con- sent forms but did not finish the survey. Most of them (1731) left the survey at the very end when asked to follow the ac- counts and upload the screenshots. These participants were not re-contacted in Wave 2 as uploading the screenshots was a necessary condition to finish the survey and be eligible for Wave 2. The distribution of these participants across condi- tions is similar to the distribution of participants who finished the first wave. There is no sign of differential attrition across Controls/Treatments.<br>Appendix I.2. Germany<br>In Germany, in Wave 1, a total of 8009 participants took the survey.<br>1420 participants did not pass the initial screens and were excluded at the very beginning of the survey because they did not report having a WhatsApp or an Instagram account (225), reported never using WhatsApp and Instagram (149), or were already following one of the social media accounts (1046).<br>2859 participants passed the screens but voluntarily ended the survey on the second consent form, when being told that they would have to follow two social media accounts on What- sApp/Instagram for two weeks. Most of these participants re- ported not wanting to follow new accounts on social media fortwo weeks – and only 15% reported that the compensation was too low.<br>1721 participants passed the screens and filled out both con- sent forms but did not finish the survey. Most of them (1611) left the survey at the very end when asked to follow the ac- counts and upload the screenshots. These participants were not re-contacted in Wave 2 as uploading the screenshots was a nec- essary condition to finish the survey. The distribution of these participants across Control/Treatment is similar to the distribu- tion of participants who finished the first wave. There is no sign of differential attrition across Controls/Treatments. However, participants in the WhatsApp conditions were more likely to drop out than participants in the Instagram conditions. Suggest- ing that in Germany participants may have struggled to follow the accounts on WhatsApp. Such differential attrition between WhatsApp/Instagram groups is not problematic for causal in- ference given that it does not impede the Controls/Treatments randomization – i.e., the WhatsApp Treatments are compared to the WhatsApp Controls. |
| Ethics oversight | This research project complied with all ethical regulations for research involving human subjects and received ethical ap- proval from the University of Zu¨rich PhF Ethics Committee (ethics approval nr. 23.10.14). All study participants submit- ted informed consent before any data were collected. |

Note that full information on the approval of the study protocol must also be provided in the manuscript.

# Field-specific reporting

Please select the one below that is the best fit for your research. If you are not sure, read the appropriate sections before making your selection.

☐ Life sciences ☒ Behavioural & social sciences ☐ Ecological, evolutionary & environmental sciences

For a reference copy of the document with all sections, see nature.com/documents/nr-reporting-summary-flat.pdf

# Behavioural & social sciences study design

All studies must disclose on these points even when the disclosure is negative.

| | |
|---|---|
| Study description | Quantitative. It's a two wave survey, and in between waves we implemented a field experiment by requiring participants to follow two accounts on social media |
| Research sample | In 2024, between March 1 and March 11, we recruited 2,009 German and 2,021 French participants via the mar- ket research company Bilendi. Participants were recontacted between March 18 and 28, 1,700 German participants (985 women, mean age = 40.5 (12.5), median education = A two- year college degree, 38% had a bachelor degree or more), and 1,695 French participants completed the second wave (1,042 women, mean age = 44.1 (11.5), median education = finished high school, 31% had a bachelor degree or more). The median distance between waves was 14 days (M = 13.4, SD = 1.74). InAppendix I we report all exclusions. |
| Sampling strategy | Random. Sample size was determined by budget mostly |
| Data collection | Online, via Qualtrics |
| Timing | see above |
| Data exclusions | see above |
| Non-participation | see above |
| Randomization | Participants first consented to take part in the study and an- swered screening questions about (i) whether they have Insta- gram and WhatsApp accounts, (ii) how frequently they use them, and (iii) whether they follow a list of social media ac- counts. To be eligible, participants had to have either an In- stagram account or WhatsApp, use Instagram or WhatsApp, and not already be following the news accounts included in the experiment. After passing the screens, Instagram users were randomly assigned to the Instagram Control or the Instagram Treatment, WhatsApp users were randomly assigned to the WhatsApp Control or the WhatsApp Treatment, and those who used both Instagram and WhatsApp were randomly assigned to one of the four conditions (see Figure 1). |

# Reporting for specific materials, systems and methods

We require information from authors about some types of materials, experimental systems and methods used in many studies. Here, indicate whether each material, system or method listed is relevant to your study. If you are not sure if a list item applies to your research, read the appropriate section before selecting a response.

## Materials & experimental systems

| n/a | Involved in the study |
|---|---|
| ☒ | ☐ Antibodies |
| ☒ | ☐ Eukaryotic cell lines |
| ☒ | ☐ Palaeontology and archaeology |
| ☒ | ☐ Animals and other organisms |
| ☒ | ☐ Clinical data |
| ☒ | ☐ Dual use research of concern |
| ☒ | ☐ Plants |

## Methods

| n/a | Involved in the study |
|---|---|
| ☒ | ☐ ChIP-seq |
| ☒ | ☐ Flow cytometry |
| ☒ | ☐ MRI-based neuroimaging |

## Plants

| | |
|---|---|
| Seed stocks | *Report on the source of all seed stocks or other plant material used. If applicable, state the seed stock centre and catalogue number. If plant specimens were collected from the field, describe the collection location, date and sampling procedures.* |
| Novel plant genotypes | *Describe the methods by which all novel plant genotypes were produced. This includes those generated by transgenic approaches, gene editing, chemical/radiation-based mutagenesis and hybridization. For transgenic lines, describe the transformation method, the number of independent lines analyzed and the generation upon which experiments were performed. For gene-edited lines, describe the editor used, the endogenous sequence targeted for editing, the targeting guide RNA sequence (if applicable) and how the editor was applied.* |
| Authentication | *Describe any authentication procedures for each seed stock used or novel genotype generated. Describe any experiments used to assess the effect of a mutation and, where applicable, how potential secondary effects (e.g. second site T-DNA insertions, mosiacism, off-target gene editing) were examined.* |
